# A Three-Dimensional Coordination Framework with a Ferromagnetic Coupled Ni(II)-CrO_4_ Layer: Synthesis, Structure, and Magnetic Studies

**DOI:** 10.3390/polym14091735

**Published:** 2022-04-24

**Authors:** Hsu-Yen Tang, Gene-Hsiang Lee, Kwai-Kong Ng, Chen-I Yang

**Affiliations:** 1 Department of Chemistry, Tunghai University, Taichung 407, Taiwan; eunice5332@gmail.com; 2 Instrumentation Center, National Taiwan University, Taipei 106, Taiwan; ghlee@ntu.edu.tw; 3 Department of Applied Physics, Tunghai University, Taichung 407, Taiwan

**Keywords:** magnetic coordination polymer, chromate-bridge, pillar-layered network, ferromagnetic, antiferromagnetic, magnetic ordering, metamagnetism

## Abstract

We report herein on the crystal structure and magnetic studies of a three-dimensional (3D) Ni(II)-chromate coordination polymer, [Ni(CrO_4_)(bpym)(H_2_O)]*_n_* (**1**; bpym = 5,5′-bipyrimidin), prepared by self-assembly of Ni(II) and chromate ions with a multi-*N* donor auxiliary ligands, bpym, through hydrothermal processes. The structure of **1** is composed of Ni(II)-CrO_4_ layers with [Ni_3_(*μ*_3_-CrO_4_)] triangular motifs, in which the Ni(II) centers are bridged by *O*′:*O*′:*O*′:*μ*_3_-CrO_4_^2−^ anions, and the resulting layers are further connected by twisted *trans*-*μ*_2_-*N*,*N*′-bpym auxiliary ligands to form a 3D pillar-layered network with an **hms** topology. The magnetic properties of compound **1** were illustrated by variable field and temperature magnetic susceptibility measurements. The findings reveal that compound **1** shows intralayer ferromagnetic interactions within Ni(II)-CrO_4_ layers, and furthers the 3D antiferromagnetic ordering in the resulting of interlayer antiferromagnetic couplings with a Néel temperature (*T*_N_) of 5.6 K. In addition, compound **1** shows the field-induced metamagnetic behavior at temperature below the *T*_N_.

## 1. Introduction

The recent reports on the design and preparation of novel coordination polymers (CPs), crystalline materials that contain both organic and inorganic moieties through coordination bonds, have attracted great attention in the field of material science [1,2,3,4,5,6,7,8,9,10,11,12,13,14]. Due to the hybrid properties of the inorganic–organic structures, these types of CPs include a variety of architectures and topologies. Because of the fascinating properties of these materials, they have the potential for use in ion exchange applications, gas storage, luminescence, and heterogeneous catalysis [15,16,17,18,19]. Although CPs such as these have many other potential applications, the development of magnetic CPs for molecular magnetism has become an important area of interest [20,21,22,23,24]. To design multi-dimensional magnetic CPs that show specific and tunable properties, the selection of appropriate bridging ligands defectively mediate the magnetic interactions between the spin carriers is important [25,26]. Therefore, extensive efforts have been made to develop magnetic CPs that contain potentially useful bridging ligands that have the affinity to coordinate metal ions for the construction of various extended frameworks that have desirable magnetic properties.

Tetrahedral oxyanions, such as SO_4_^2−^, SeO_4_^2−^, PO_4_^3^^−^, and A_S_O_4_^3−^, are extensively employed as short bridging groups, because they not only have the ability to connect multiple metal ions to produce extended structures by their extreme versatile coordination modes, but also are able to mediate significant magnetic interactions, thus conferring diverse magnetic properties, such as ferromagnetism, spin-canted antiferromagnetism, magnetic ordering, and metamagnetism [27,28,29,30]. Although the CrO_4_^2−^ anion also has been used as bridge in the synthesis of many CPs due to the similarity of its various bonding mode, only few metal-chromate magnetic CPs have been reported in literature [31,32,33,34]. On the other hand, the general approach for the preparation of multi-dimensional magnetic CPs has involved the bonding of some coordination sites of the metal centers with suitable auxiliary ligands, while also allowing co-ligands to be connected to the remaining coordination vacancies.

Therefore, in the synthetic strategy described in this report, we utilized a bipyrimidyl ligand, 5,5′-bipyrimidine (bpym), as an auxiliary ligand. The characteristics of this ligand include: (i) the bpym ligand contains four N-donor sites for connecting multiple metal centers to extend the structure; (ii) the versatile geometrical structure of the bpym caused by out of the plane orientation of two pyrimidyl rings at different dihedral angles confers a sufficient flexibility and adaptability to permit coordination polymers with diverse frameworks to be constructed; and (iii) the N donor-rich backbones may aid in the formation of supramolecular structures in which hydrogen bonding and π-π interactions are sufficient to stabilize the multi-dimensional structure.

We herein report on the preparation and characterization of a new 3D Ni(II)-chromate coordination polymer, [Ni(CrO_4_)(bpym)(H_2_O)]*_n_* (**1**), using bpym and CrO_4_^2−^ as co-ligands. Compound **1** adopts 3D a pillar-layered framework composed by Ni(II)-CrO_4_ inorganic layers and bpym pillars. Magnetic investigations revealed that compound **1** exhibits intralayer Ni(II)···Ni(II) ferromagnetic coupling by the chromate bridges, and the layers are further antiferromagnetically coupled in the low-temperature range resulting in an antiferromagnetic ordering below *T*_N_ = 5.6 K. Furthermore, a field-induced magnetic phase transition of metamagnetism below its *T*_N_ was observed for compound **1.**

## 2. Experimental

### 2.1. Materials and Methods 

Reagents and solvents were purchased commercially and used as received. 5,5′-bipyrimidine was synthesized following the literature method [35].

### 2.2. Synthesis of [Ni(CrO_4_)(bpym)(H_2_O)]_n_ (1)

A mixture of Ni(SO_4_)_2_·6H_2_O (15.5 mg, 0.10 mmol), bpym (15.8 mg, 0.10 mmol), and K_2_CrO_4_ (19.4 mg, 0.10 mmol) was added to 6 mL of H_2_O in a 25 mL Teflon-lined Parr acid digestion reactor. The reactor was sealed, heated to 100 °C for 72 h, and then gradually cooled to 23 °C at a rate of 1 °C per hour. Yellow crystals of **1** (29.9 mg, 85% yield based on bpym) suitable for X-ray analysis were obtained, washing with distilled water, collecting with suction filtration, and drying in air. The bulk samples show a well compared pattern with simulation of single-crystal one. Anal. Calcd (found) for C_8_H_8_CrN_4_NiO_5_ (**1**): C, 27.38; H, 2.30; N, 15.97. Found: C, 27.20; H, 2.18; N, 15.98. IR data (KBr disk, cm^−1^): 3391(w), 3066(w), 1646(m),1586(m), 1567(m), 1410(s), 1352(m), 1193(m), 1138(w), 1059(w), 1018(s), 940(w), 926(w), 908(w), 883(m), 716(m), 690(w), 655(m).

### 2.3. X-ray Crystallography

Diffraction data of compound **1** was collected on a Brucker APEX3 CCD diffractometer (Bruker, Karlsruhe, Germany) with graphite-monochromated Mo-Kα radiation (λ = 0.7107 Å). The data collection temperature was controlled at 150 K with open flow of N_2_ cryostreams. Empirical absorption corrections were applied using the SADABS program [36]. The SHELXTL-2014 program was used to solve and to refine the structure by direct methods and the full-matrix least-squares method, respectively [37]. The thermal factor of all non-hydrogen atoms was refined anisotropically. Atoms of C4, C8, and N4 in one of the pyrimidyl rings were disordered over two positions with the refined occupancies of 0.543 and 0.457 for C4, C8, and N4 and C4′, C8′, and N4′, respectively. The hydrogen atoms of the coordinated water molecule were located from differential electron density maps, whereas the hydrogen atoms of bpym ligand were constrained to the ideal positions riding on their parent atoms with isotropic thermal parameters. A summary of data of compound **1** is presented in Table 1, and selected bond lengths and angles are given in Table 2.

### 2.4. Physical Measurements

IR spectra were recorded on a spectrometer of Perkin-Elmer Spectrum RX1 FTIR using KBr pellets (Elementar, Langenselbold, Germany). Elemental analysis of C, H, and N was carried out with an Elemental varioEL III elemental analyzer (Seiko Instruments, Chiba shi, Japan). Thermogravimetric (TG) analysis was performed on an EXSTAR 6200 TG/DTA thermal analyzer (Seiko Instrumental, Inc.) under nitrogen atmosphere at a 5 °C/min heating rate. Powder X-ray diffraction data were collected on a Rigaku D/max 2000 diffractometer (Rigaku Instruments, Tokyo, Japan) at room temperature with Cu-Kα (λ = 1.5406 Å) radiation. DC (direct current) and AC (alternating current) magnetic susceptibilities of microcrystalline samples of compound **1** were performed on a Quantum Design MPMS-7 SQUID and a PPMS magnetometer, respectively (Quantum Design, San Diego, CA, USA). Experimental magnetic susceptibility data were corrected by the diamagnetic contributions estimated from a background of the sample holder and Pascal’s constants [38].

## 3. Results and Discussion

### 3.1. Syntheses and Characterization of Compound **1**

Compound **1** was successfully prepared under hydrothermal conditions by the reaction of NiSO_4_ salts with the K_2_CrO_4_ ligand and the bpym ligand with a 1:1:1 of Ni:CrO_4_^2−^ bpym molar ratio in H_2_O at 100 °C. The phase purity of a bulk sample of **1** was further confirmed by X-ray powder diffraction (XRPD) patterns and elemental analysis. The measured XRPD patterns of compound **1** are in good agreement with patterns simulated from the single-crystal data (Figure S1 in the Supporting Information). The thermal stability of compound **1** was also characterized by TG analysis (Figure S2), in which the TG curve of **1** shows that the removal of water proceeds in one step, resulting in a total weight loss of 5.5% in the temperature range of 205–240 °C, corresponding to the loss of the coordinated water molecules (calcd, 5.1%). The host framework begins to decompose and undergoes rapid weight loss at temperatures above ~320 °C, which can be attributed to the decomposition of the bpym ligand. In the IR spectrum, strong and broad absorption peaks around 880–800 cm^−1^ are assigned to the Cr–O vibrations, the strong bands in the range 1650–1450 cm^−1^ are assigned to the vibrations of C=C and C=N groups in the bpym.

### 3.2. Description of Structure

#### Crystal Structures Description of [Ni(CrO_4_)(bpym)(H_2_O)]_n_ (**1**)

**Scheme 1:**
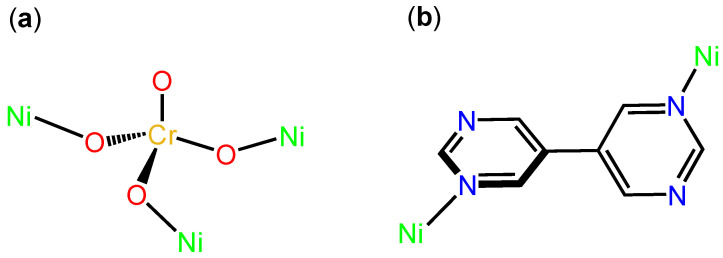
(**a**) The *μ*_3_-*O*,*O*′,*O*”-CrO_4_^2−^; (**b**) the twisted *trans*-*μ*_2_-*N*,*N*′-bpym in compound **1**.

**Figure 1:**
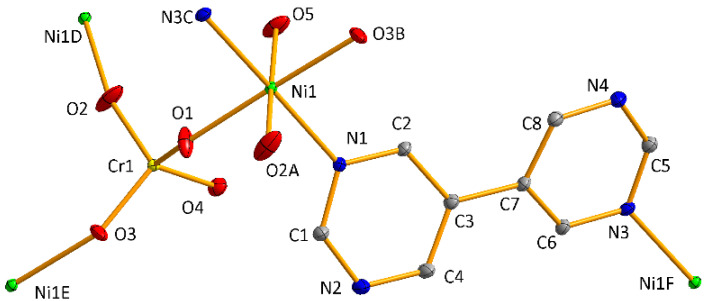
The coordination environments of compound **1**. The H atoms have been omitted for clarity. (Symmetry code: (**A**) 1 − x, −0.5 + y, 2−z; (**B**) 1 + x, y, z; (**C**) x, y, 1 + z; (**D**) 1 − x, −0.5 + y, 2 − z; (**E**) 1 + x, y, z; (**F**) x, y, −1 + z). **A**–**E** refer symmetrical cords of atom in Figure 1, which are labled as O2A, O3B, Ni1D, Ni1E, and Ni1F.

**Figure 2:**
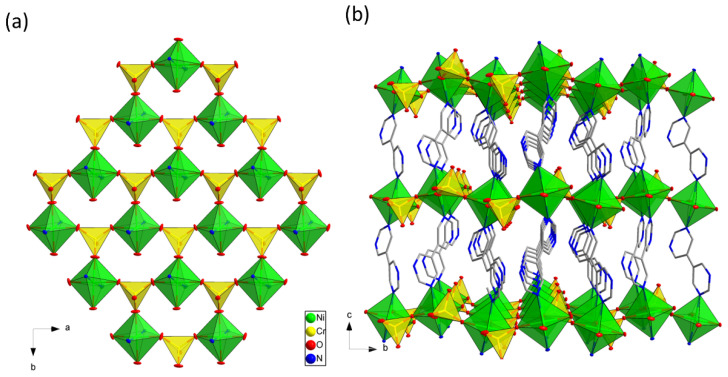
(**a**) Polyhedral view of the Ni(II)-CrO_4_ inorganic layer along the *c* axis, and (**b**) the 3D pillar-layered structure of compound **1** along the *b* axis. The NiN_2_O_4_ and CrO_4_ sites are shown as green octahedrons and yellow tetrahedrons, respectively.

A single-crystal X-ray structure diffraction analysis reveals that **1** is crystallized in monoclinic with a chiral *P*2_1_ space group and possesses 3D frameworks comprised of Ni(II)-CrO_4_ inorganic layers pillared by bpym ligands (Figure 1). In compound **1**, the asymmetric unit contains one crystallographically independent Ni(II) ion, one chromate anion, one coordinated water molecule, and one bpym ligand. As shown in Figure 1a, the Ni(II) ions adopt a distorted *trans*-NiN_2_O_4_ octahedral geometry made up of four equatorial oxygen atoms from three bridging CrO_4_^2^^−^ groups (O1, O2, and O3) and one coordinated water molecule (O5), and the two axial nitrogen atoms (N1 and N3) form two bpym ligands. The Ni(II)−O/N bond lengths are in the range of 2.0242(2)–2.1219(1) Å, and the *cis* and *trans* bond angles around the Ni(II) centers of compound **1** are in the ranges of 87.18(1)–93.21(1)° and 175.52(1)–178.50(1)°, respectively. The chromate anions exhibit a slightly distorted tetrahedral coordination geometry about with O−Cr−O bond angles in the range of 107.47(13)–110.19(13)° in **1**. Each chromate anion connects three crystallographically equivalent Ni(II) ions through three of the four O atoms (O1, O2, and O3) in a rare *O*′:*O*″:*O*‴:*μ*_3_-bridging mode (Scheme 1a), whereas the Cr−O bond distances of 1.647(2), 1.6594(18), and 1.6649(19) A° for the O coordinated to Ni(II) are slightly longer than the “uncoordinated” Cr−O bond of 1.6132(18) Å. On the other hand, each Ni(II) ion is linked to six neighbors through three *cis* equatorial coordinated CrO_4_^2−^ anions, resulting in a corrugated layer with a [Ni_3_(*μ*_3_-CrO_4_)] triangular motif parallel to the *ab* crystal plane, where the uncoordinated Cr−O bond of CrO_4_^2−^ anions are located alternatively above and below the layers (Figure 2a) and the coordinated water of Ni−O bond vector is on the plane of the coordination sheet. The three unique distances of Ni(II)···Ni(II) in the layer that are spanned by the *μ*_3_-CrO_4_^2−^ bridges are 5.425, 5.529, 6.693 Å. To the best of our knowledge, such [Ni_3_(*μ*_3_-CrO_4_)]*_n_* layered structural motif has never been reported previously. In the layer, the dihedral angles between the adjacent chromate-bridged Ni(II)O_4_ equatorial planes are 0°, 22.77°, 22.77°.

The bpym ligands adopt a twisted *trans-μ*-*N*,*N*′-bridging mode (Scheme 1b; 23.95° of dihedral angle of two pyrimidyl rings), which alternating interlink the adjacent Ni(II)-CrO_4_ layers with two different orientations toward the [001] direction in the cavity-above-cavity manner, thus resulting in a pillar-layered 3D framework (Figure 2b). In addition, the framework contains the 1D channels along the *a* axis. The interlayer Ni(II)···Ni(II) separation via the bpym spacer in **1** is 9.923 Å. The shortest distance of centroid to centroid between the adjacent pyrimidyl rings of the bpym ligand in compound **1** is 4.373(1) Å, which is much higher than the *π*–*π* interactions between pyrimidyl rings. As a result, there are no significant *π*–*π* interactions found in **1**. The 3D framework is further stabilized by additional hydrogen bonding interactions involved by the coordinated water molecules and the oxygen atoms of the CrO_4_^2−^ groups. The H-bond distances are O1A…O5 = 2.821 Å, O3B…O5 = 2.884 Å, and O2C…O5 = 3.329 Å, and the angles of O1A…H5B–O5 = 129.17°, O3B…H5A–O5 = 127.28°, O2C…H5A–O5 = 145.83° (Symmetrical core: ^A^ −x + 1, y + 1/2, −z + 2; ^B^ −x + 1, y + 1/2, −z + 2, −z + 2; ^C^ −x + 1, y + 1/2, −z + 2).

**Figure 3:**
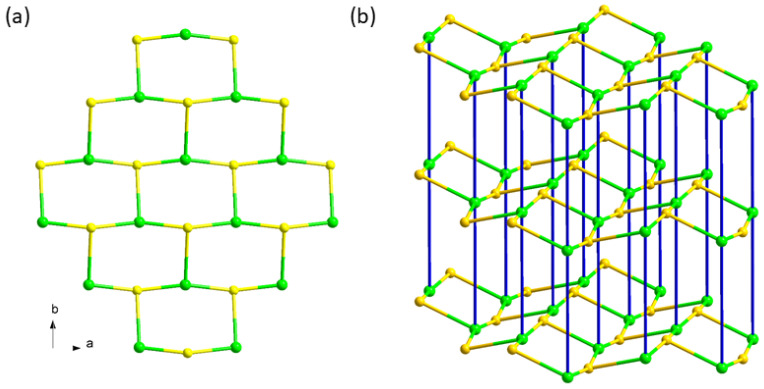
(**a**) Schematic representation of the 3-connected 2D net of Ni(II)-CrO_4_ layer, and (**b**) the binodal (3,5)-connected **hms** topology of compound **1**. The Cr, Ni centers are shown as yellow and green spheres, respectively, and bpym ligands are represented by blue sticks.

The analysis of topology of the 3D framework of compound **1** indicates that each octahedral Ni(II) is linked by three *μ*_3_-CrO_4_^2^^−^ anions in the *cis*-positions of the equatorial planes and two twist *trans*-*μ*_2_-*N*,*N*′-bpym ligands in its axial positions, which can be viewed as a 5-connected node through omitting the terminal coordinated H_2_O molecules in the equatorial positions, while each *μ*_3_-CrO_4_^2^^−^ anion and each bpym ligand can be considered to be a triangular 3-connected node and a 2-connector. Such connectivity repeats are infinite and to give the Ni(II)-CrO_4_ layer and 3D network of **1** as schematically shown in Figure 3. Analysis by the TOPOS software package shows that the framework of compound **1** can be described as a binodal (3,5)-connected **hms** topology with the Schläfli symbol (6^3^)(6^9^.8) [39].

### 3.3. Magnetic Properties

**Figure 4:**
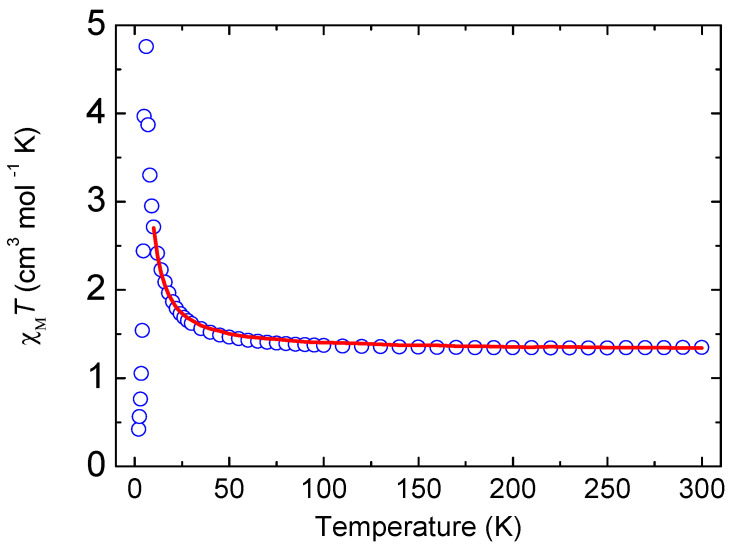
The temperature dependence of *χ*_M_*T* for compound **1** at temperatures between 2 to 300 K with a 1 kOe applied field. The solid line shows the best fit of the data mentioned in the text.

The thermal variation of magnetic susceptibility for compound **1** was collected from 2 to 300 K under a 1000 Oe magnetic field and shown as *χ*_M_*T* and *χ*_M_ per Ni(II) ion in Figure 4 and Figure S3. The room-temperature *χ*_M_*T* value of compound **1** is 1.35 cm^3^ K mol^−1^, which was found to be appreciably higher than the theoretical value (1.00 cm^3^ K mol^−1^ with *g* = 2.0) for a spin-only Ni(II) ion. The *χ*_M_*T* value increases gradually upon cooling, then increases rapidly at temperatures below 30 K, reaching a maximum of 4.76 cm^3^ K mol^−1^ at 6.0 K, implying the existence of ferromagnetic couplings. At temperatures above 40 K, the data of magnetic susceptibility obey the Curie–Weiss law with *C* = 1.32 cm^3^ mol^−1^ K and *θ* = 3.93 K (Figure S4). The positive value of *θ* indicates the presence of ferromagnetic coupling between the near-neighbor Ni(II) ions. Upon further cooling, the *χ*_M_*T* value abruptly decreases to 0.42 cm^3^ mol^−1^ K at 2.0 K, suggesting overall antiferrmagneticism in the low-temperature range.

In attempting to understand the nature of the magnetic coupling between the Ni(II) ions of the compound **1** by fitting the magnetic susceptibility data, some assumptions were made by taking the structure into further magnetic account. In the first step, the interlayer magnetic coupling through the bpym ligands was ignored due the long Ni···Ni bridging distance. In the second step, two different magnetic couplings (*J*_1_ and *J*_2_) were assumed for the [Ni_3_(*μ*_3_-CrO_4_)]-based layer by considering the similarity of Ni···Ni distances and Ni–O–O–Ni torsion angles, where *J*_1_ are magnetic coupling through CrO_4_^2^^−^ with Ni···Ni separations of 5.425 and 5.529 Å and *J*_2_ is the magnetic coupling through CrO_4_^2^^−^ with a Ni···Ni separation of 6.693 Å. Therefore, compound **1** can be considered to be a Heisenberg isosceles triangular layer, as shown in Figure S5, and the isotropic exchange Hamiltonian is given as:

(1) $$\hat{H}=J_{1}\underset{\langle i,j\rangle }{\sum }\vec{S_{i}}\cdot \vec{S_{j}}+J_{2}\underset{\langle \langle i,j\rangle \rangle }{\sum }\vec{S_{i}}\cdot \vec{S_{j}}$$

The magnetic susceptibility of **1** was fitted using Monte Carlo simulations based on the Metropolis algorithm [40] in which a 40 × 40 site sample was chosen. The best fit above 10 K leads to values of *g* = 2.3 (fixed), *J*_1_ = 1.44(2) cm^−^^1^, *J*_2_ = 0.00(2) cm^−1^, and *R* = 3 × 10^−4^. In addition, a reliable fitting (*R* = 3.6 × 10^−4^) with parameters of *g* = 2.3 (fixed), *J*_1_ = 1.53 cm^−1^, and *J*_2_ = −0.01 cm^−1^ was also observed, which indicates that the very small value for *J*_2_ cannot be determined precisely in the fitting. The fitting results indicate ferromagnetic interaction (*J*_1_) between Ni(II) ions through CrO_4_^2^^−^ bridged along the *a* and *b* axes, and a neglectable magnetic exchange (*J*_2_) between the Ni(II) ions through the CrO_4_^2^^−^ bridged along the *a* axis, giving a [Ni_3_(*μ*_3_-CrO_4_)]-based ferromagnetic layer. The ferromagnetic nature of the magnetic coupling of *J*_1_ is in good agreement with values for related Ni–CrO_4_ compounds [27,28,30], which can be explained by an accidental orthogonality between the *σ*-type frontier orbitals of the *t*_2_ type molecular orbitals of the CrO_4_^2^anion and $d_{x^{2}-y^{2}}$ orbitals of the Ni(II) ions, while the neglectable magnetic interaction of *J*_2_ can be attributed to the long Ni–O–Cr–O–Ni bridging distance.

In order to investigate the magnetic behavior in the low-temperature range, variable-temperature zero-field cooling and field cooling (ZFC/FC) magnetizations and temperature dependence alternating current (AC) magnetic susceptibility were measured. As shown in Figure S6, in a weak of 10 Oe applied field, upon cooling, the ZFC/FC magnetization increases gradually reaching a sharp maximum at 5.4 K, and then rapidly drops to 2 K without any divergence, indicative of antiferromagnetic ordering evoked by weak interlayer antiferromagnetic interactions. Figure S7 shows plots of the temperature-dependent in-phase (χ_M_′) and out-of-phase (χ_M_″) signals of the AC magnetic susceptibility for compound **1**, in which the frequency independent χ_M_’ signal exhibits a peak located at 5.6 K without a noticeable χ_M_’’ signal, thus confirming the occurrence of antiferromagnetic ordering below the Néel temperature (*T*_N_) at 5.6 K. This is consistent with results obtained for ZFC/FC magnetization.

**Figure 5:**
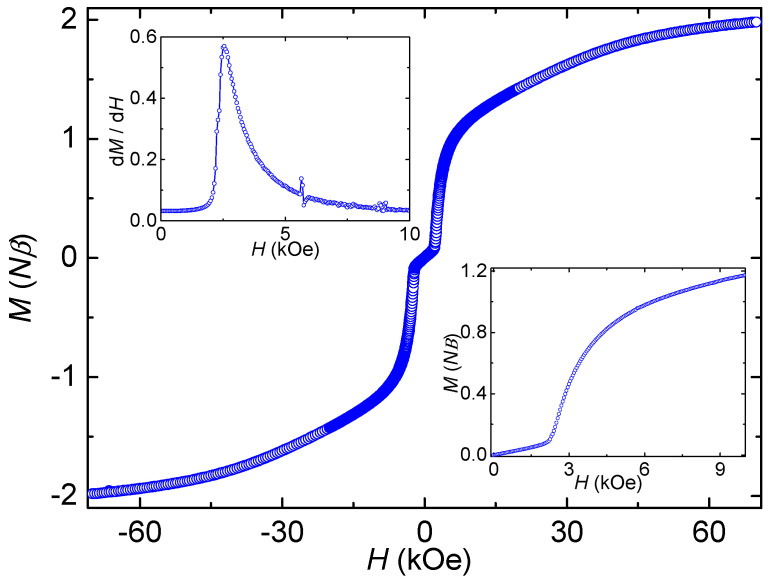
The isothermal field-dependent magnetization of **1** at 1.8 K. Insets: The low-field *M*-*H* and *dM/dH* plots.

Furthermore, the isothermal field-dependence magnetization up to 70 kOe for compound **1** was measured at 1.8 K and the results are shown in Figure 5. The cure of magnetization (*M*) showed a distinct sigmoid shape, in which the value for *M* increases slowly and linearly in the field below ~2.5 kOe, and abruptly increases in the region of 2.5–4.0 kOe, before slowly increasing again to reach a maximum value of 2.02 *Nβ* at 70 kOe, which is slightly lower than the expected saturation value of 2.3 *Nβ* for an Ni(II) system with *g* = 2.3. The sigmoid shape of *M* clearly implies the existence of field-induced metamagnetism, in which the antiparallel alignments of net moments by weak antiferromagnetic couplings are overcome by a critical external field, which is accompanied by a magnetic state transition from an antiferromagnetic state (*AF*) to a paramagnetic state (*P*). The slow increase of magnetization at the field below ~2.5 kOe implies the presence of antiferromagnetic couplings between the ferromagnetic coupled Ni(II)-CrO_4_ layer layers, which are likely mediated by dipolar interactions due to the long spacing of the bpym bridges. In contrast, the rapid increase of magnetization at the field above ~2.5 kOe suggests an overcoming of interlayer antiferromagnetic couplings by the applied field. At 1.8 K, the critical field (*H*_C_) of magnetic phase transition of compound **1** is around 2.55 kOe by estimated of *dM*/*dH* (Figure 5, up-inset). In the literature, the metamagnetic behavior has been reported in layered or chain compounds showing competing magnetic interactions and strong magnetic anisotropy [41,42,43,44,45,46,47,48]. As we can see, a narrow hysteresis loop can be detected between the field in the region of 2.4–3.7 kOe (Figure S8).

**Figure 6:**
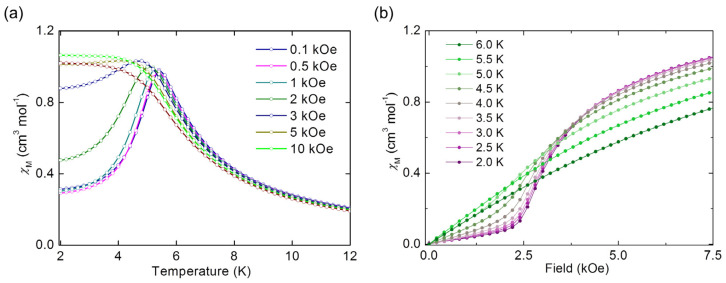
(**a**) The plots of FC magnetization below 12 K at various fields, and (**b**) the plots of field-dependent magnetization under different temperatures of compound **1**.

**Figure 7:**
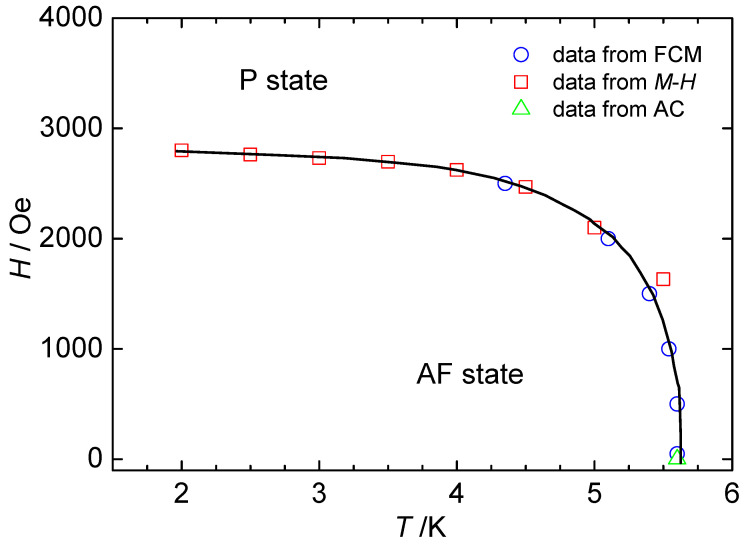
Temperature vs. field magnetic phase diagram of **1**. The open blue cycles, open red squares, and open green triangle are data from the locations of maximum in FCM data, *dM*/*dH* vs. *H* data, and *χ*_M_′ in ac data; the solid line is to guide the eye.

Such information of metamagnetic transition prompted more temperature dependence of FC magnetizations, *χ*_M_(*T*), at several applied fields, and more field dependence magnetizations, *M*(*H*), at different temperatures for *H-T* magnetic phase diagram. As can be seen in Figure 6a, the peaks of the *χ*_M_(*T*) curves are field-dependent, which shift to lower temperatures as increasing field. As the applied field larger than 3.0 kOe, the peaks of *χ*_M_(*T*) disappear and become a plateau. Figure 6b shows *M*(*H*) curves collected in the field region of 0–7.5 kOe at different temperatures, in which the step-wise shaped abnormality of *M*(*H*) curves becomes pronounced and vanishes as temperatures increase from 2.0 to 6.0 K, which can be seen clearly in the differentials of these data (Figure S9). To combine the FCM and *M*(*H*) data, the *H*-*T* magnetic phase diagram of compound **1** was obtained and shown in Figure 7, which signifies a field-induced *AF* to P state transition of the metamagnetic behavior in the compound **1** below its antiferromagnetic ordering temperature.

## 4. Conclusions

A new pillared-layer 3D framework composed of Ni(II) ions, CrO_4_^2−^ anions, and a bpym ligand as the nitrogen donor. The structure of compound **1** is composed of Ni(II)-CrO_4_ layers, formed from triangular[Ni_3_(*μ*_3_-CrO_4_)]*_n_* motifs, and linear *μ*_2_-bpym pillars with a **hms** topology. A magnetic analysis revealed that compound **1** shows ferromagnetic interactions within the Ni(II)-CrO_4_ layer with weak antiferromagnetic coupling between the adjacent layers, resulting in an overall antiferromagnetic ordering, and exhibits field-induced metamagnetism. The findings reported here demonstrate that chromate is particularly promising for use in the synthesis of multi-dimensional coordination polymers with versatile structural topologies and special magnetic properties.

## Figures and Tables

**Table 1:** Crystallographic data for compound **1**.

Formula	C_8_H_8_CrN_4_NiO_5_
Fw	350.89
Crystal system	Monoclinic
Space group	*P*2_1_
*a*/Å	6.6931(2)
*b*/Å	8.4975(2)
*c*/Å	9.8328(2)
α/°	90
β/°	102.7114(9)
γ/°	90
*V*/Å^3^	545.53(2)
Z	2
*T*/K	150(2)
*D*_c_/g cm^−3^	2.136
*μ*/mm^−1^	2.745
*R*_int_	0.0255
*θ*_max_/°	34.983
Goodness-of-fits on *F*^2^	1.030
*R*_1_^1^, w*R*_2_^2^ (all data)	0.0274, 0.0611
*R*_1_^1^, w*R*_2_^2^ (I > 2σ (I))	0.0255, 0.0598
Flack	0.026(13)

^1^*R*_1_ (Σ||*F*_O_| − |*F*_C_||)/Σ|*F*_O_|; ^2^ w*R*_2_ = [Σw|*F*_O_^2^ − *F*_C_^2^|^2^/Σw(*F*_O_^4^)]^1/2^.

**Table 2:** Selected bond distances (Å) and angles (°) for compounds **1**.

Compound 1			
Ni(1)-O(2) ^1^	2.0233(19)	Ni(1)-O(5)	2.087(2)
Ni(1)-O(1)	2.0242(17)	Ni(1)-N(1)	2.0998(19)
Ni(1)-O(3) ^2^	2.0505(17)	Ni(1)-N(3) ^3^	2.122(2)
Cr(1)-O(4)	1.6132(18)	Cr(1)-O(2)	1.647(2)
Cr(1)-O(3)	1.6594(18)	Cr(1)-O(1)	1.6649(19)
O(3)2-Ni(1)-N(1)	88.17(7)	O(1)-Ni(1)-N(3) ^3^	89.57(8)
O(5)-Ni(1)-N(1)	92.04(8)	O(3)^2^-Ni(1)-N(3) ^3^	90.88(7)
O(2)^1^-Ni(1)-N(3) ^3^	87.18(8)	O(5)-Ni(1)-N(3) ^3^	92.32(8)
O(2)^1^-Ni(1)-O(1)	88.20(11)	N(1)-Ni(1)-N(3) ^3^	175.51(8)
O(2)^1^-Ni(1)-O(3) ^2^	89.96(10)	Cr(1)-O(1)-Ni(1)	134.85(13)
O(1)-Ni(1)-O(3) ^2^	178.08(11)	Cr(1)-O(2)-Ni(1) ^4^	159.65(14)
O(2)^1^-Ni(1)-O(5)	178.50(11)	Cr(1)-O(3)-Ni(1) ^5^	145.98(14)
O(1)-Ni(1)-O(5)	93.21(10)	C(2)-N(1)-Ni(1)	122.84(15)
O(3)^2^-Ni(1)-O(5)	88.64(9)	C(1)-N(1)-Ni(1)	120.28(16)
O(2)^1^-Ni(1)-N(1)	88.44(8)	C(5)-N(3)-Ni(1) ^6^	119.9(5)
O(1)-Ni(1)-N(1)	91.25(8)	C(6)-N(3)-Ni(1) ^6^	121.83(15)

Symmetry transformations used to generate equivalent atoms: ^1^ −x + 1, y − 1/2, −z + 2; ^2^ x + 1, y, z; ^3^ x, y, z + 1; ^4^ −x + 1, y + 1/2, −z + 2; ^5^ x − 1, y, z; ^6^ x, y, z − 1.

## Data Availability

The data presented in this study are available in the manuscript and Supplementary Material.

## Supplementary Materials

The following are available online at https://www.mdpi.com/article/10.3390/polym14091735/s1, Figure S1: Simulated PXRD pattern (red) and experimental PXRD pattern (black) of compound **1**, Figure S2: Thermogravimetric (TG) analysis diagram of compound **1**, Figure S3: Plot of χ_M_ (○) vs. *T* for a powdered sample of compound **1**, Figure S3: Plot of χ_M_^−1^ (○) vs. T of compound **1**. The solid line represents the best fit χ_M_^−1^ above 500 K with a Curie-Weiss law, Figure S4: Plot of χ_M_ (○) vs. T of compound **1**, Figure S5: Magnetic exchange coupling scheme of [Ni_3_(*μ*_3_-CrO_4_)]-based layer of compound **1**, Figure S6: ZFC/FC magnetizations of compound **1** at the field of 10 Oe, Figure S7: In phase (χ′) and out-off phase (χ′′) of the AC magnetic susceptibilities in a zero applied DC field and a 3.5 G AC field at the indicated frequencies for compound **1**, Figure S8: A blow-up of the hysteresis loop of compound 1 at the 1.8 K, Figure S9: d*M*/d*H* vs. *H* plots for the virgin magnetization of compound **1**.
