# Supplementary material for: A Three-Dimensional Coordination Framework with a Ferromagnetic Coupled Ni(II)-CrO4 Layer: Synthesis, Structure, and Magnetic Studies
Source: Polymers (Basel). 2022 Apr 24;14(9):1735. doi: 10.3390/polym14091735 (PMC9103387; doi:10.3390/polym14091735)

checkCIF/PLATON report

Structure factors have been supplied for datablock(s) I

THIS REPORT IS FOR GUIDANCE ONLY. IF USED AS PART OF A REVIEW PROCEDURE FOR PUBLICATION, IT SHOULD NOT REPLACE THE EXPERTISE OF AN EXPERIENCED CRYSTALLOGRAPHIC REFEREE.

No syntax errors found. CIF dictionary Interpreting this report

Datablock: I

Bond precision:	C-C = 0.0033 A		Wavelength=0.71073
Cell:	a=6.6931(2)	b=8.4975(2)	c=9.8328(2)
	alpha=90	beta=102.7114(9)	gamma=90
Temperature:	150 K		

	Calculated	Reported
Volume	545.53(2)	545.53(2)
Space group	P 21	P 21
Hall group	P 2yb	P 2yb
Moiety formula	C8 H8 Cr N4 Ni O5	?
Sum formula	C8 H8 Cr N4 Ni O5	C8 H8 Cr N4 Ni O5
Mr	350.87	350.89
Dx,g cm-3	2.136	2.136
Z	2	2
Mu (mm-1)	2.745	2.745
F000	352.0	352.0
F000'	353.45	
h,k,lmax	10,13,15	10,13,15
Nref	4800[2530]	4778
Tmin,Tmax	0.743,0.931	0.774,0.901
Tmin'	0.697	

Correction method= # Reported T Limits: Tmin=0.774 Tmax=0.901
AbsCorr = MULTI-SCAN

Data completeness= 1.89/1.00 Theta(max)= 34.983

R(reflections)= 0.0255(4577) wR2(reflections)= 0.0611(4778)

S = 1.030 Npar= 183

The following ALERTS were generated. Each ALERT has the format
test-name_ALERT_alert-type_alert-level.
Click on the hyperlinks for more details of the test.

Alert level B

```
PLAT430_ALERT_2_B Short Inter D...A Contact  O2       ..N3       .       2.86 Ang.
                                              1-x,1/2+y,1-z  =      2_656 Check
```

Alert level C

```
PLAT048_ALERT_1_C MoietyFormula Not Given (or Incomplete) .......     Please Check
PLAT314_ALERT_2_C Small Angle for H2O: Metal-O5       -H5A       .      93.57 Degree
PLAT430_ALERT_2_C Short Inter D...A Contact  O2       ..O3       .       2.88 Ang.
                                               -x,1/2+y,2-z  =      2_557 Check
PLAT761_ALERT_1_C CIF Contains no X-H Bonds .....................     Please Check
PLAT762_ALERT_1_C CIF Contains no X-Y-H or H-Y-H Angles ..........     Please Check
PLAT911_ALERT_3_C Missing FCF Refl Between Thmin & STh/L=    0.600          6 Report
PLAT976_ALERT_2_C Check Calcd Resid. Dens.  0.73Ang From O5      .      -0.41 eA-3
PLAT977_ALERT_2_C Check Negative Difference Density on H5B       .      -0.33 eA-3
```

Alert level G

```
PLAT003_ALERT_2_G Number of Uiso or Uij Restrained non-H Atoms ...          4 Report
PLAT004_ALERT_5_G Polymeric Structure Found with Maximum Dimension          3 Info
PLAT007_ALERT_5_G Number of Unrefined Donor-H Atoms .............          2 Report
PLAT019_ALERT_1_G _diffrn_measured_fraction_theta_full/*_max < 1.0      0.996 Report
PLAT171_ALERT_4_G The CIF-Embedded .res File Contains EADP Records          3 Report
PLAT186_ALERT_4_G The CIF-Embedded .res File Contains ISOR Records          1 Report
PLAT301_ALERT_3_G Main Residue  Disorder .............(Resd  1  )        16% Note
PLAT432_ALERT_2_G Short Inter X...Y Contact  O3       ..C5'      .       2.99 Ang.
                                                 -1+x,y,1+z  =      1_456 Check
PLAT432_ALERT_2_G Short Inter X...Y Contact  O4       ..C6       .       2.96 Ang.
                                              1-x,1/2+y,1-z  =      2_656 Check
PLAT860_ALERT_3_G Number of Least-Squares Restraints ............         25 Note
PLAT883_ALERT_1_G No Info/Value for _atom_sites_solution_primary .     Please Do !
PLAT910_ALERT_3_G Missing # of FCF Reflection(s) Below Theta(Min).          1 Note
PLAT913_ALERT_3_G Missing # of Very Strong Reflections in FCF ....          2 Note
PLAT933_ALERT_2_G Number of HKL-OMIT Records in Embedded .res File          1 Note
PLAT941_ALERT_3_G Average HKL Measurement Multiplicity ..........        3.0 Low
PLAT967_ALERT_5_G Note: Two-Theta Cutoff Value in Embedded .res ..       70.0 Degree
PLAT978_ALERT_2_G Number C-C Bonds with Positive Residual Density.          4 Info
```

0 **ALERT level A** = Most likely a serious problem - resolve or explain
1 **ALERT level B** = A potentially serious problem, consider carefully
8 **ALERT level C** = Check. Ensure it is not caused by an omission or oversight
17 **ALERT level G** = General information/check it is not something unexpected

5 ALERT type 1 CIF construction/syntax error, inconsistent or missing data
10 ALERT type 2 Indicator that the structure model may be wrong or deficient
6 ALERT type 3 Indicator that the structure quality may be low
2 ALERT type 4 Improvement, methodology, query or suggestion
3 ALERT type 5 Informative message, check

checkCIF publication errors

Alert level A

```
PUBL004_ALERT_1_A  The contact author's name and address are missing,
            _publ_contact_author_name and _publ_contact_author_address.
PUBL005_ALERT_1_A  _publ_contact_author_email, _publ_contact_author_fax and
            _publ_contact_author_phone are all missing.
            At least one of these should be present.
PUBL006_ALERT_1_A  _publ_requested_journal is missing
            e.g. 'Acta Crystallographica Section C'
PUBL008_ALERT_1_A  _publ_section_title is missing. Title of paper.
PUBL009_ALERT_1_A  _publ_author_name is missing. List of author(s) name(s).
PUBL010_ALERT_1_A  _publ_author_address is missing. Author(s) address(es).
PUBL012_ALERT_1_A  _publ_section_abstract is missing.
            Abstract of paper in English.
```

Alert level G

```
PUBL017_ALERT_1_G The _publ_section_references section is missing or
            empty.
```

---

7 **ALERT level A** = Data missing that is essential or data in wrong format
1 **ALERT level G** = General alerts. Data that may be required is missing

---

Publication of your CIF

You should attempt to resolve as many as possible of the alerts in all categories. Often the minor alerts point to easily fixed oversights, errors and omissions in your CIF or refinement strategy, so attention to these fine details can be worthwhile. In order to resolve some of the more serious problems it may be necessary to carry out additional measurements or structure refinements. However, the nature of your study may justify the reported deviations from journal submission requirements and the more serious of these should be commented upon in the discussion or experimental section of a paper or in the "special_details" fields of the CIF. *checkCIF* was carefully designed to identify outliers and unusual parameters, but every test has its limitations and alerts that are not important in a particular case may appear. Conversely, the absence of alerts does not guarantee there are no aspects of the results needing attention. It is up to the individual to critically assess their own results and, if necessary, seek expert advice.

If level A alerts remain, which you believe to be justified deviations, and you intend to submit this CIF for publication in a journal, you should additionally insert an explanation in your CIF using the Validation Reply Form (VRF) below. This will allow your explanation to be considered as part of the review process.

Validation response form

Please find below a validation response form (VRF) that can be filled in and pasted into your CIF.

```
# start Validation Reply Form
_vrf_PUBL004_GLOBAL
;
PROBLEM: The contact author's name and address are missing,
RESPONSE: ...
;
_vrf_PUBL005_GLOBAL
;
PROBLEM: _publ_contact_author_email, _publ_contact_author_fax and
RESPONSE: ...
;
_vrf_PUBL006_GLOBAL
;
PROBLEM: _publ_requested_journal is missing
RESPONSE: ...
;
_vrf_PUBL008_GLOBAL
;
PROBLEM: _publ_section_title is missing. Title of paper.
RESPONSE: ...
;
_vrf_PUBL009_GLOBAL
;
PROBLEM: _publ_author_name is missing. List of author(s) name(s).
RESPONSE: ...
;
_vrf_PUBL010_GLOBAL
;
PROBLEM: _publ_author_address is missing. Author(s) address(es).
RESPONSE: ...
;
_vrf_PUBL012_GLOBAL
;
PROBLEM: _publ_section_abstract is missing.
RESPONSE: ...
;
# end Validation Reply Form
```

If you wish to submit your CIF for publication in Acta Crystallographica Section C or E, you should upload your CIF via the web. If you wish to submit your CIF for publication in IUCrData you should upload your CIF via the web. If your CIF is to form part of a submission to another IUCr journal, you will be asked, either during electronic submission or by the Co-editor handling your paper, to upload your CIF via our web site.

PLATON version of 19/02/2022; check.def file version of 19/01/2022

Datablock I - ellipsoid plot

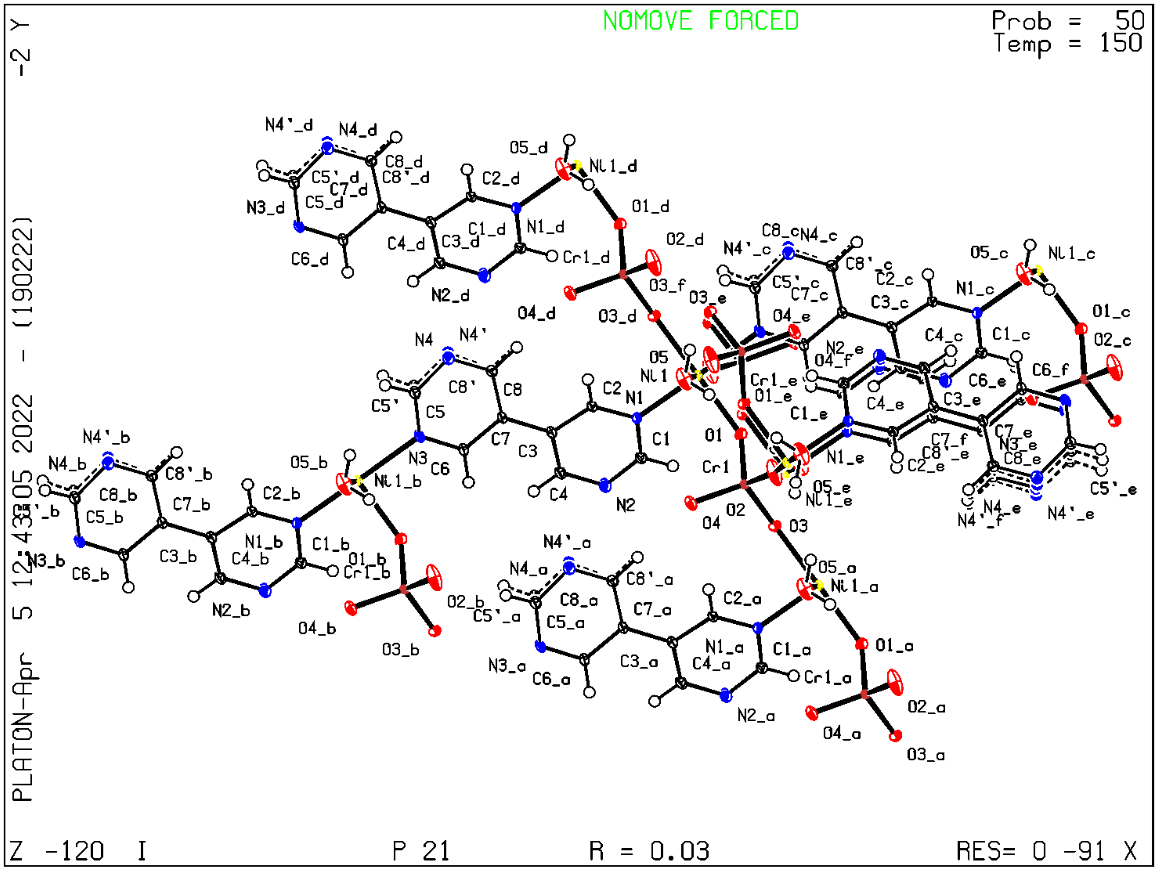

Supplement: Supplementary file 1 [file polymers-14-01735-s001.zip › checkcif.pdf]
