# Supplementary material for: A Three-Dimensional Coordination Framework with a Ferromagnetic Coupled Ni(II)-CrO4 Layer: Synthesis, Structure, and Magnetic Studies
Source: Polymers (Basel). 2022 Apr 24;14(9):1735. doi: 10.3390/polym14091735 (PMC9103387; doi:10.3390/polym14091735)
Supplement: Supplementary file 1 [file polymers-14-01735-s001.zip › Ni-CrO4-bpm_ESI_05Apr2022.pdf]

A three-dimensional coordination framework with a ferromagnetic coupled $Ni(II)-CrO_4$ layer: synthesis, structure and magnetic studies

Hsu-Yen, Tang[1], Gene-Hsiang Lee[2], Kwai-Kong Ng[3,*], and Chen-I Yang[1,*]

[1]Department of Chemistry, Tunghai University, Taichung 407, Taiwan
[2]Instrumentation Center, National Taiwan University, Taipei 106, Taiwan
[3]Department of Applied Physics, Tunghai University, Taichung 407, Taiwan

	Index	Page
**Figure S1.**	Simulated PXRD pattern (red) and experimental PXRD pattern (black) of compound **1.**	S2
**Figure S2.**	Thermogravimetric (TG) analysis diagram of compound **1**.	S3
**Figure S3.**	Plot of χ_M (○) *vs. T* for a powdered sample of compound **1**.	S4
**Figure S4.**	Plot of χ_M^{-1} (○) *vs.* T for a powdered sample of compound **1**. The solid line represents the best fit χ_M^{-1} above 50 K with a Curie–Weiss law.	S5
**Figure S5.**	Magnetic exchange coupling scheme of $[Ni_3(\mu_3\text{-}CrO_4)]$-based layer of compound **1**.	S6
**Figure S6.**	ZFC/FC magnetization plots of compound **1** at the field of 10 G.	S7
**Figure S7.**	In phase (χ') and out-off phase (χ'') of the ac magnetic susceptibilities in a zero applied dc field and a 3.5 G ac field at the indicated frequencies for compound **1**.	S7
**Figure S8.**	A blow-up of the hysteresis loop of compound **1** at the 1.8 K.	S8
**Figure S9.**	d*M*/d*H vs. H* plots for the virgin magnetization of compound **1**.	S8

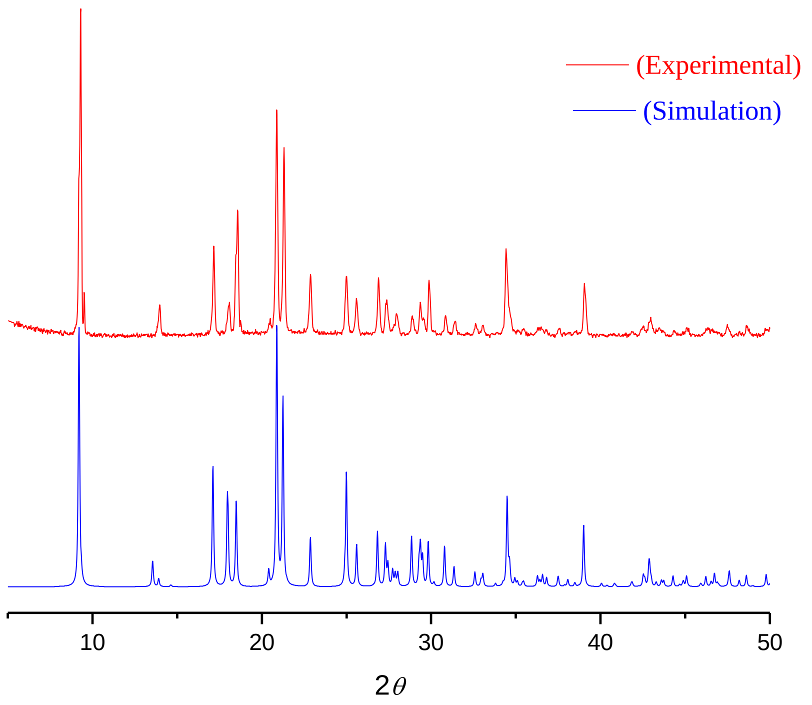

Figure. S1. Simulated PXRD pattern (red) and experimental PXRD pattern (black) of compound **1**.

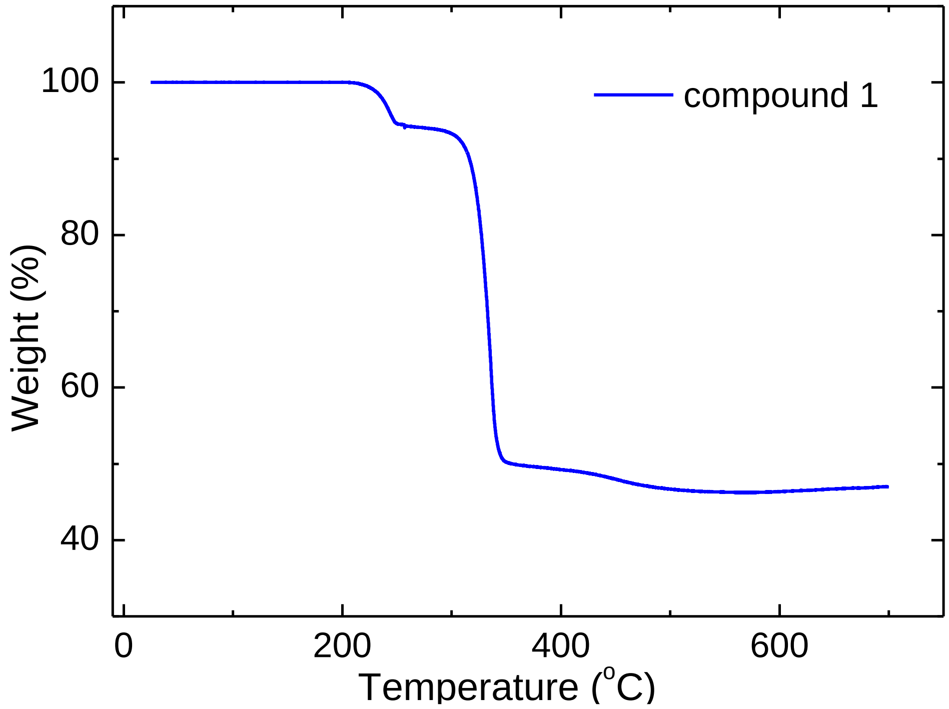

Figure S2. Thermogravimetric (TG) analysis diagram of compound **1**.

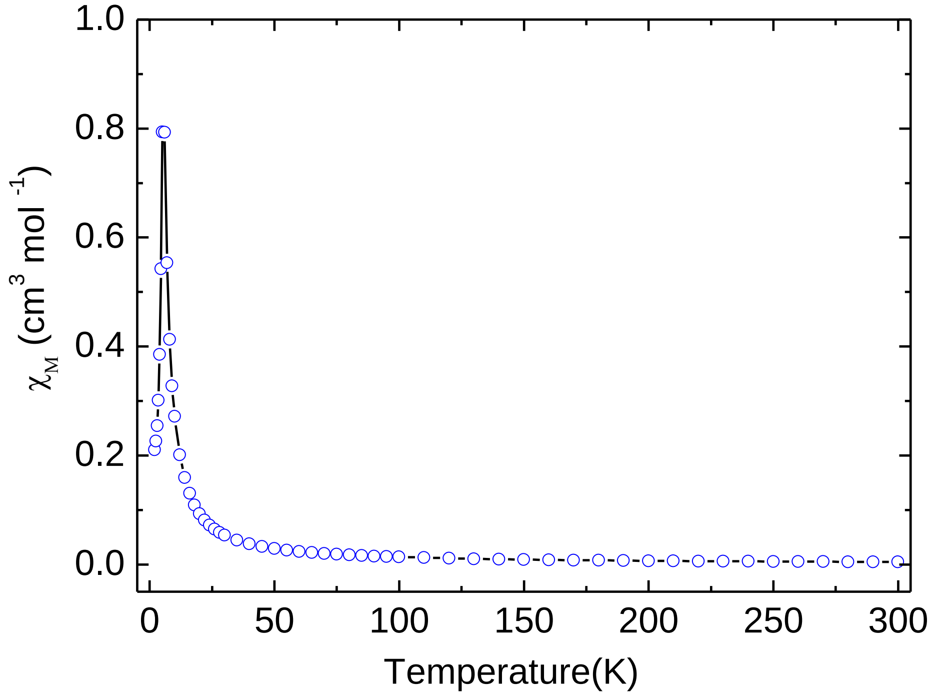

Figure S3. Plot of χ_M (○) *vs.* *T* for a powdered sample of compound **1**.

.

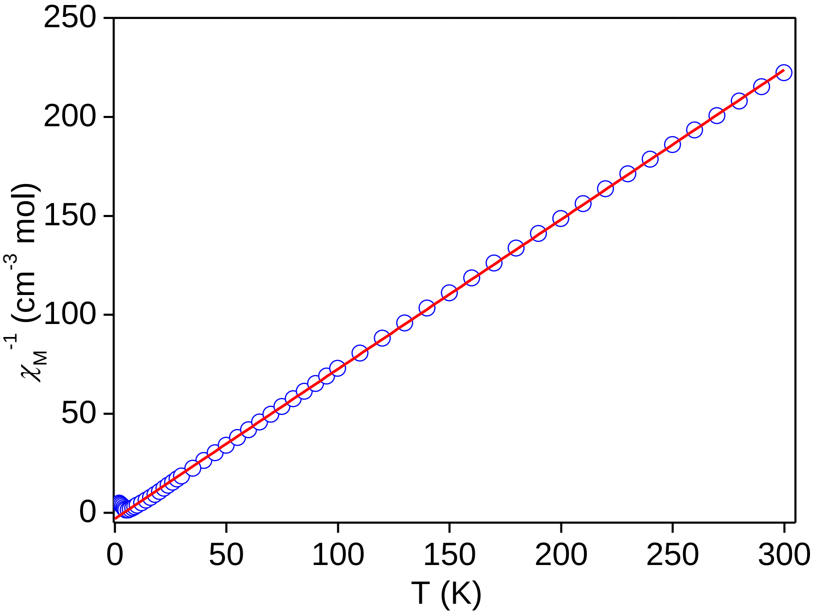

Figure S4. Plot of χ_M^{-1} (○) *vs.* T for a powdered sample of compound **1**. The solid line represents the best fit χ_M^{-1} above 40 K with a Curie–Weiss law.

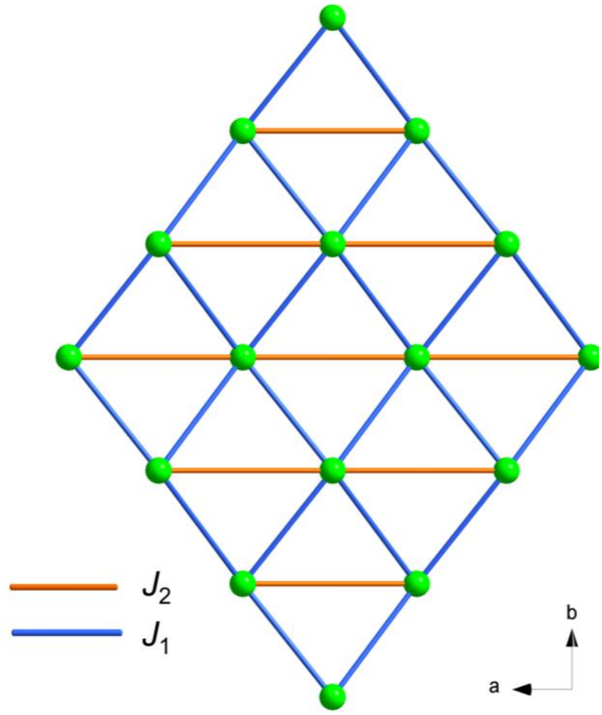

Figure S5. Magnetic exchange coupling scheme of [Ni_3(μ_3-CrO_4)]-based layer of compound **1**.

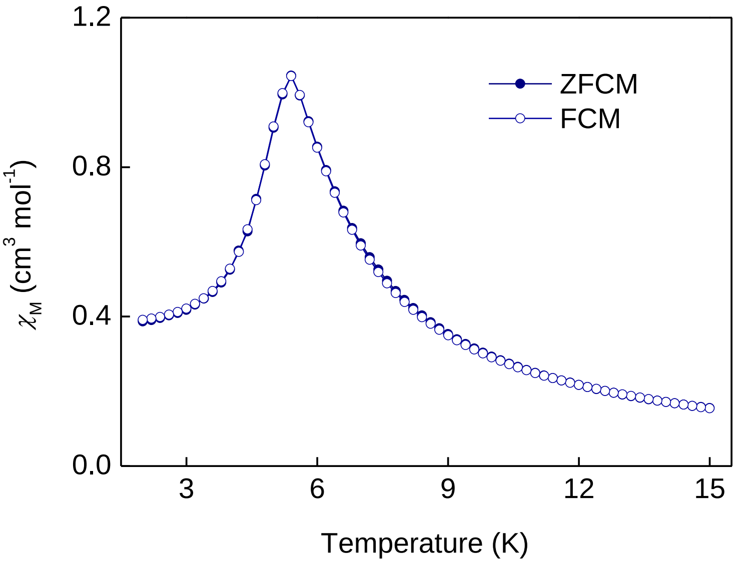

Figure S6. ZFC/FC magnetization plots of compound **1** at the field of 10 G.

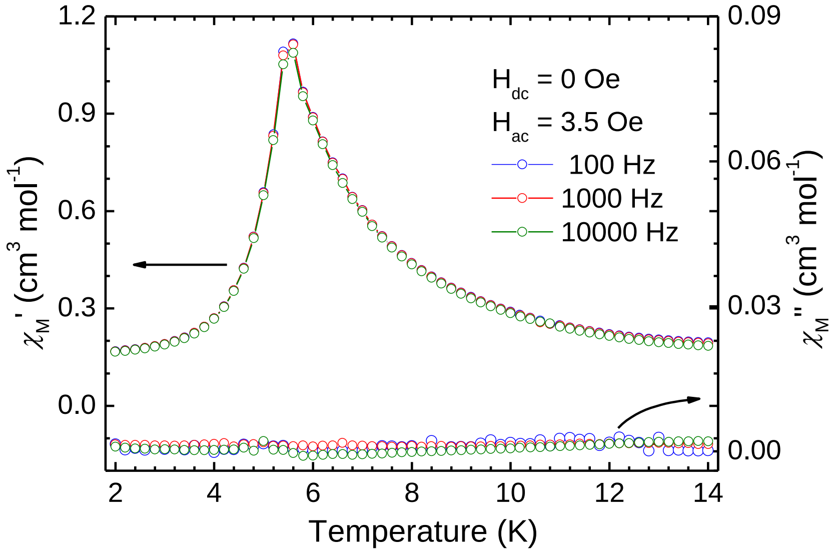

Figure S7. In phase (χ′) and out-off phase (χ″) of the ac magnetic susceptibilities in a zero applied dc field and a 3.5 G ac field at the indicated frequencies for compound **1**.

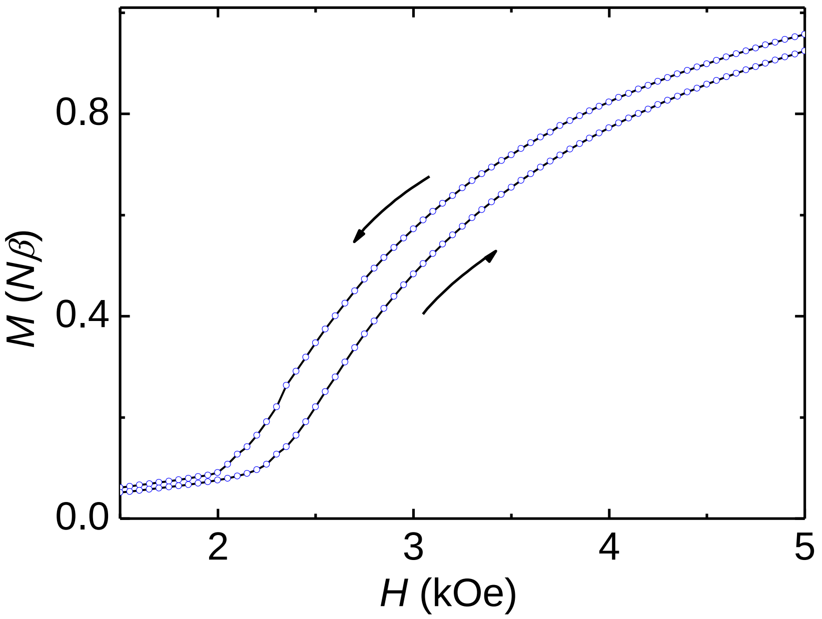

Figure S8. A blow-up of the hysteresis loop of compound **1** at the 1.8 K.

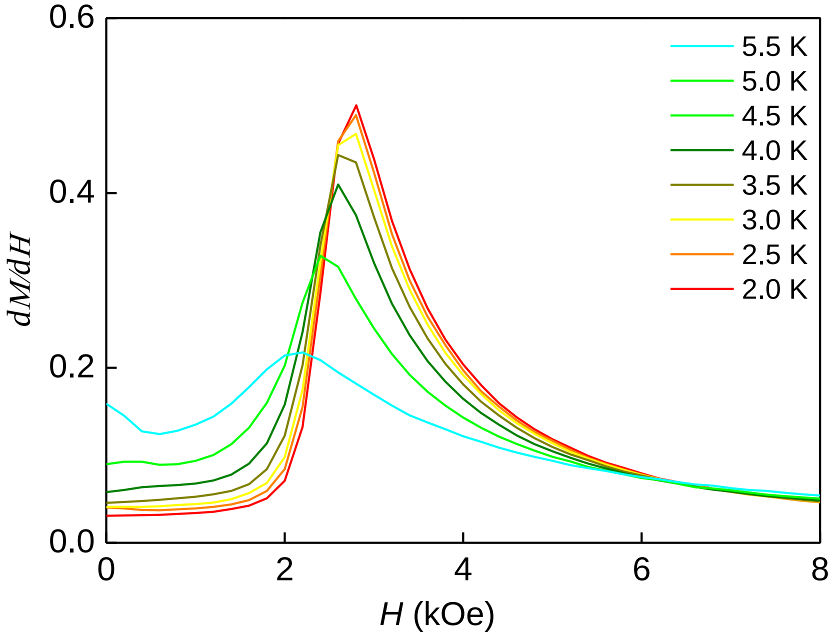

Figure S9. dM/dH *vs.* H plots for the virgin magnetization of compound **1**.
